# Multidrug Resistance-Associated Protein 2 Deficiency Aggravates Estrogen-Induced Impairment of Bile Acid Metabolomics in Rats

**DOI:** 10.3389/fphys.2022.859294

**Published:** 2022-03-21

**Authors:** Fatemeh Alaei Faradonbeh, Hana Lastuvkova, Jolana Cermanova, Milos Hroch, Zuzana Nova, Martin Uher, Petra Hirsova, Petr Pavek, Stanislav Micuda

**Affiliations:** ^1^Department of Pharmacology, Faculty of Medicine in Hradec Kralove, Charles University, Hradec Kralove, Czechia; ^2^Department of Medical Biochemistry, Faculty of Medicine in Hradec Kralove, Charles University, Hradec Kralove, Czechia; ^3^Division of Gastroenterology and Hepatology, Mayo Clinic, Rochester, MN, United States; ^4^Department of Pharmacology and Toxicology, Faculty of Pharmacy in Hradec Kralove, Charles University, Hradec Kralove, Czechia

**Keywords:** Mrp2-deficient rats, estrogen, cholestasis, bile acids, Nrf2

## Abstract

Multidrug resistance-associated protein 2 (Mrp2) mediates biliary secretion of anionic endobiotics and xenobiotics. Genetic alteration of Mrp2 leads to conjugated hyperbilirubinemia and predisposes to the development of intrahepatic cholestasis of pregnancy (ICP), characterized by increased plasma bile acids (BAs) due to mechanisms that are incompletely understood. Therefore, this study aimed to characterize BA metabolomics during experimental Mrp2 deficiency and ICP. ICP was modeled by ethinylestradiol (EE) administration to Mrp2-deficient (TR) rats and their wild-type (WT) controls. Spectra of BAs were analyzed in plasma, bile, and stool using an advanced liquid chromatography–mass spectrometry (LC–MS) method. Changes in BA-related genes and proteins were analyzed in the liver and intestine. Vehicle-administered TR rats demonstrated higher plasma BA concentrations consistent with reduced BA biliary secretion and increased BA efflux from hepatocytes to blood *via* upregulated multidrug resistance-associated protein 3 (Mrp3) and multidrug resistance-associated protein 4 (Mrp4) transporters. TR rats also showed a decrease in intestinal BA reabsorption due to reduced ileal sodium/bile acid cotransporter (Asbt) expression. Analysis of regulatory mechanisms indicated that activation of the hepatic constitutive androstane receptor (CAR)-Nuclear factor erythroid 2-related factor 2 (Nrf2) pathway by accumulating bilirubin may be responsible for changes in BA metabolomics in TR rats. Ethinylestradiol administration to TR rats further increased plasma BA concentrations as a result of reduced BA uptake and increased efflux *via* reduced *Slco1a1* and upregulated Mrp4 transporters. These results demonstrate that Mrp2-deficient organism is more sensitive to estrogen-induced cholestasis. Inherited deficiency in Mrp2 is associated with activation of Mrp3 and Mrp4 proteins, which is further accentuated by increased estrogen. Bile acid monitoring is therefore highly desirable in pregnant women with conjugated hyperbilirubinemia for early detection of intrahepatic cholestasis.

## Introduction

Estrogen-induced cholestasis is regarded clinically as the hepatic disorder in women with increased estrogen levels during pregnancy, or it may develop during estrogen administration as a part of hormonal contraception or hormonal replacement therapy (Rezai et al., 2015). Increased estrogen production contributes to the development of intrahepatic cholestasis of pregnancy (ICP), which is characterized by symptoms including pruritus, abnormal liver function, and raised serum bile acid (BA) levels, occurring especially in the third trimester. Besides unpleasant subjective symptoms, ICP threatens the fetus with a higher incidence of adverse pregnancy outcomes such as iatrogenic preterm delivery, nonreassuring fetal status, meconium staining of the amniotic fluid, and stillbirth. Prophylaxis and effective therapy of ICP are therefore of the highest priority. The need for proper management of ICP is further accentuated by a significant overall incidence of this disorder. The main factor responsible for fetal injury during ICP is elevated BAs, especially when plasma BAs concentration exceeds 40 mM. Therefore, understanding the factors which may predispose to or alleviate the accumulation of BAs during ICP is currently at the center of attention. Furthermore, individual BAs show different characteristics, with hydrophobic ones, such as lithocholic acid or deoxycholic acid, being more toxic than hydrophilic species, such as ursodeoxycholic acid (UDCA), which is even used as first-line therapy in ICP. These BAs also express different potency and efficacy to activate BA receptors, such as farnesoid X receptor (FXR) and pregnane X receptor (PXR). Spectra of BAs must be therefore analyzed to understand pathophysiological consequences.

Multidrug resistance-associated protein 2 (Mrp2) is a major apical efflux pump for biliary secretion of various amphipathic organic anion conjugates, including BAs. Mrp2 creates a key component of BA-independent bile formation by mediating biliary secretion of glutathione (GSH). Homozygous mutations of the gene encoding Mrp2 (ABCC2) cause Dubin-Johnson syndrome, a rare liver disorder that presents with conjugated hyperbilirubinemia (Jemnitz et al., 2010). In contrast, BA metabolomics has not been entirely studied in individuals with Mrp2 deficiency. Douglas et al. (1980) initially reported increased fasting conjugated cholate concentration and prolonged intravenous clearance of sodium glycocholate in a woman with Dubin-Johnson syndrome. Reduced cholic acid (CA) clearance was also detected in sheep exhibiting inherited defects in hepatic bilirubin transport similar to human Dubin-Johnson syndrome (Engelking and Gronwall, 1979). More recent work reports that net plasma concentrations of BAs are often increased in individuals with Mrp2 mutations (Togawa et al., 2018; Junge et al., 2021), although exceptions also exist (Fu et al., 2021). To date, individual BAs in individuals with Mrp2 mutations have not been analyzed. Moreover, several studies have provided evidence that ABCC2 variants are associated with an increased risk of ICP and cholestasis induced by estrogen contraceptives (Sookoian et al., 2008; Dixon et al., 2017; Kularatnam et al., 2017; Huynh et al., 2018; Corpechot et al., 2020), albeit a contradictory report also exists (Meier et al., 2008). The exact mechanisms whereby Mrp2 deficiency modifies BA metabolomics and its relationship to ICP have been poorly studied.

Given this knowledge gap, the present study aimed to elucidate the role of Mrp2 in the development of estrogen-induced cholestasis. We used Mrp2-deficient rats together with their wild-type (WT) controls and induced cholestasis by repeated administration of ethinylestradiol (EE). Previous experimental studies failed to find any alteration of BA biliary secretion in Mrp2-deficient rats after a single dose or 3-day EE regimen (Koopen et al., 1998; Huang et al., 2000). However, impairment of bile flow during ICP is more chronic. Therefore, we used the current standard model of ICP that involves ethinylestradiol administration over a 5-day period. This longer ethinylestradiol treatment worsened cholestasis in Mrp2-deficient rats and significantly altered BA metabolomics accompanied by increased plasma BA concentrations.

## Materials and Methods

### Chemicals

Ethinylestradiol (>98% purity), methanol, acetonitrile, ammonium acetate, acetic acid, formic acid (each in LC/MS grade purity), and D5 CA were purchased from Merck (Prague, Czech Republic). Bile acid standards were purchased from Steraloids, Inc. (Newport, Rhode Island) and Sigma-Aldrich (St. Louis, Missouri).

### Animal Study

Multidrug resistance-associated protein 2 deficient (TR, transporter-deficient) Lewis rats or complementary Lewis WT rats were a kind gift from Prof. Ingrid Kloting, Institut für Pathophysiologie, Karlsburg, Germany. All experimental protocols were conducted in accordance with EU Directive 2010/63/EU for animal experiments. The project was approved by the Animal Welfare Bodies of the Faculty of Medicine in Hradec Kralove and the Ministry of Education, Youth and Sports of the Czech Republic (Approval No. MSMT-23573/2015-5). Animals were housed at a constant humidity of 55 ± 10% and a constant temperature of 22 ± 1°C under a 12 h light/dark cycle with free access to water and food. Around 12-week-old female TR and WT rats were randomized to receive either propanediol (vehicle) or EE (5 mg/kg body weight) subcutaneously once daily for 5 consecutive days (six animals/group). Therefore, there were four groups of animals: (i) WT-Ve – WT rats administered with the vehicle; (ii) WT-EE – WT rats receiving ethinylestradiol; (iii) TR-Ve – TR rats administered with the vehicle; and (iv) TR-EE – TR rats receiving ethinylestradiol. The stool was collected over the last 24-h after the last administration of the ethinylestradiol to analyze bile acid fecal elimination. Thereafter, all animals underwent a clearance study performed under general anesthesia induced by pentobarbital (50 mg/kg, i.p.). Herein, the bile duct was cannulated and bile was collected for 60 min. Sample of blood for bile acid analysis was taken in the middle of the collection period from the cannulated carotid artery. A blood sample for biochemical analysis was taken after bile collection. Animals were then sacrificed by anesthetic overdose, and organs were removed and stored at −80°C for further analysis.

### Analytical Methods

Standard biochemical analyses of plasma samples from rats were performed by routine methods in Central laboratories of University Hospital using Modular PP analyzer (Roche, Basel, Switzerland). The analysis of bile acids was performed using the Acquity I-Class UHPLC system (Waters, Milford, United States), implementing separation on YMC Triart C18 column 50 × 2.1 mm (YMC, Japan). The gradient separation was accomplished at flow rate of 0.35 ml.min^−1^ and temperature 45°C with mobile phase composed of solvent A (0.5 mM ammonium acetate, acetic acid 0.001% v/v) and solvent B (methanol:acetonitrile – 75:25 v/v mixture with 0.5 mM ammonium acetate and 0.001% v/v acetic acid). Gradient program was as follows: 0–0.2 min, 40% of solvent B; 0.2–7.0 min, 40–70% of solvent B; 7.0–8.0 min, 70–90% of solvent B; 8.0–8.5 min, 90–95% of solvent B; and 9.0–11 min 40% of solvent B. Xevo-TQ/XS triple quadrupole (Waters, Milford, United States) operated in negative ESI mode was used for detection. Compounds were monitored using multiple reaction monitoring transitions: 375→375 (non-conjugated monohydroxy BA), 391→391 (non-conjugated dihydroxy BA), 407→407 (non-conjugated trihydroxy BA), 432→74 (glycine-conjugated monohydroxy BA), 448→74 (glycine-conjugated dihydroxy BA), 464→74 (glycine-conjugated trihydroxy BA), 482→80 (taurine-conjugated monohydroxy BA), 498→80 (taurine-conjugated dihydroxy BA), and 514→80 (taurine-conjugated trihydroxy BA). Ion source settings were as follows: Capillary voltage 2.5 kV, cone voltage 50 V, desolvation temperature 600°C, desolvation gas 1,000 L/h, and cone gas 350 L/h. MassLynx software was used for LC/MS data acquisition (Version 4.2, Waters, Milford, United States). The concentrations of individual BA were summed to calculate the concentration of conjugated, unconjugated, and total BA. Primary BA: (T/G)CA [(tauro/glyco)cholic acid], (T)CDCA [(tauro)chenodeoxycholic acid], (T)αMCA, and (T)βMCA [(tauro)muricholic acid]; secondary BA: (T)DCA [(tauro)deoxycholic acid], (T)LCA [(tauro)lithocholic acid], (T)UDCA [(tauro)ursodeoxycholic acid], (T)MDCA [(tauro)murideoxycholic acid], THCA (taurohyocholic acid), (T)HDCA [(tauro)hyodeoxycholic acid]; 12α-OH BA: (T)CA and (T)DCA; non12α-OH BA refers to all the remaining BA. Concentrations of reduced GSH and oxidized glutathione (GSSG) were analyzed using a validated HPLC method with fluorescence detection, as described previously (Hirsova et al., 2013).

### Quantification of Gene and Protein Expression Levels

Quantitative reverse transcription-PCR (qRT-PCR) and Western blot analyses were completed using previously reported methods (Prasnicka et al., 2017). Individual gene assays are listed in Table 1 and were purchased from Thermo Fisher Scientific (MA, United States). Antibodies used for Western blot are summarized in Table 2.

**Table 1:** Pre-designed TaqMan^®^ Gene Expression Assay kits (Life Technologies) used for quantitative reverse transcription-PCR (qRT-PCR).

Gene symbol	Protein (when different name from gene)	Life technologies assay ID
*Slc10a1*	Ntcp	Rn00566894_m1
*Abcb11*	Bsep	Rn00582179_m1
*Cyp7a1*		Rn00564065_m1
*Cyp8b1*		Rn00579921_s1
*Cyp27a1*		Rn00710298_m1
*Cyp2c22*		Rn01410778_m1
*Abcc2*	Mrp2	Rn00563231_m1
*Abcc3*	Mrp3	Rn01452854_m1
*Abcc4*	Mrp4	Rn01465702_m1
*Oatp1*	Slco1a1	Rn00755148_m1
*Oatp2*	Slco1a4	Rn00756233_m1
*Oatp4*	Slco1b2	Rn00668623_m1
*Abcg2*	Bcrp	Rn00710585_m1
*Gclc*		Rn00689046_m1
*Gpx2*		Rn00822100_gH
*Nqo1*		Rn00566528_m1
*Asbt*	Slc10a2	Rn00691576_m1
*Fgf15*	Fgf19	Rn00590708_m1
*Shp*	Nr0b2	Rn00589173_m1
*GAPD* (*GAPDH*)	4352338E	Rn99999916_s1
*Euk 18S rRNA*	4333760F	Hs99999901_s1

**Table 2:** Primary and secondary antibodies used in Western blot.

Protein	Source (cat. number)	Dilution	Secondary antibody dilution
*Mrp2*	Enzo (ALX-801-037-C125)	1:300	1:2,000
*Mrp3*	Thermo Fisher (PA5–101482)	1:1,000	1:2,000
*Mrp4*	Cell signaling (12857S)	1:1,000	1:2,000
*Cyp27a1*	Thermo Fisher (PA5-27946)	1:1,000	1:2,000
*Cyp7a1*	Sigma Aldrich (MABD42)	1:1,000	1:2,000
*Cyp8b1*	Thermo Fisher (PA5–37088)	1:1,000	1:2,000
*Cyp2c70*	MyBioSource (MBS3223844)	1:1,000	1:2,000
*Ntcp*	Sigma-Aldrich (SAB2108757)	1:1,000	1:2,000
*Bsep*	Thermo Fisher (PA5-78690)	1:000	1:2,000
*p-SAPK/JNK*	Cell signaling (4668S)	1:1,000	1:2,000
*p-Erk 1/2* (*P-P44/42*)	Cell signaling (4370S)	1:1,000	1:2,000
*RXRa*	Cell signaling (5388S)	1:1,000	1:2,000
*P65_NFKB*	Abcam (ab16502)	1:1,000	1:2,000
*Gapdh*	Cell signaling (2118)	1:8,000	1:10,000

### Statistical Analysis

All statistical analyses were performed using the statistical software GraphPad Prism 6 (San Diego, United States). The results were initially tested for distribution to select the appropriate tests. The data are presented as medians with boxes and whiskers representing the interquartile range and 5th–95th percentiles, respectively. The statistical significance (*p* < 0.05) was determined using either one-way ANOVA for continuous variables or one-way ANOVA on ranks for nonparametric variables.

## Results

### Mrp2 Deficiency Alters Plasma Biochemical Parameters

**Figure 1:**
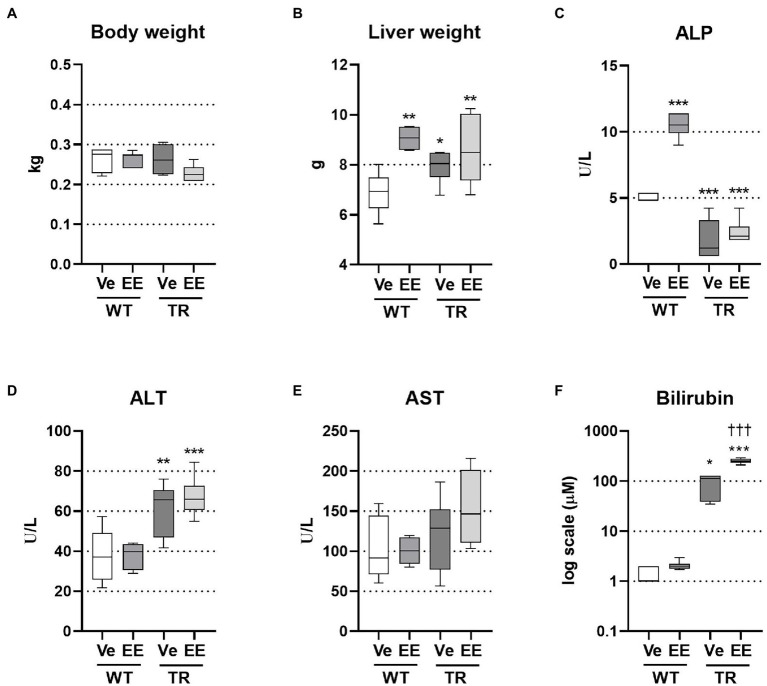
Ethinylestradiol (EE)-induced cholestasis exacerbates hyperbilirubinemia in multidrug resistance-associated protein 2 (Mrp2)-deficient TR rats. Cholestasis was induced by EE administration (5 mg/kg, s.c.) over 5 consecutive days. Experimental groups: WT-Ve, WT rats receiving vehicle; WT-EE, WT rats receiving ethinylestradiol; TR-Ve, TR rats receiving vehicle; and TR-EE, TR rats receiving ethinylestradiol. **(A)** Body weight at the end of the experiment. **(B)** Liver weight. **(C)** Plasma alkaline phosphatase (ALP) activity. **(D)** Plasma alanine aminotransferase (ALT) activity. **(E)** Plasma aspartate aminotransferase (AST) activity. **(F)** Plasma bilirubin concentrations. Data are presented as medians, with boxes and whiskers representing the interquartile range and 5th-95th percentiles, respectively. Significance: ^*^p < 0.01, ^**^p < 0.01, ^***^p < 0.001 compared to vehicle-treated wild-type (WT) rats; ^†††^*p* < 0.001 compared to vehicle-treated TR rats.

To study Mrp2 function during cholestasis, we employed TR rats, which harbor a spontaneous mutation in the Abcc2 gene leading to premature codon termination and Mrp2 deficiency. To induce cholestasis, female TR rats and WT controls received repeated doses of EE. Mrp2 deficiency or ethinylestradiol treatment did not affect the final bodyweight, while liver weight was significantly increased by ethinylestradiol or Mrp2 deficiency (Figures 1A,B). Plasma alkaline phosphatase (ALP) activity, a marker of cholestatic injury, was significantly increased by ethinylestradiol, but decreased in TR rats (Figure 1C). This unexpected reduction of plasma ALP activity in TR rats may be a consequence of negative interference of the detection method with high bilirubin concentrations (Wang et al., 2014). Mrp2 deficiency increased hepatocellular injury as evident by increased plasma alanine aminotransferase (ALT) activity, though increase in aspartate aminotransferase (AST) did not reach statistical significance (Figures 1D,E). Administration of ethinylestradiol did not increase activities of plasma transaminases in either group, which is commonly reported in rodent models of EE-induced cholestasis (Pozzi et al., 2003; Crocenzi et al., 2006; Zhao et al., 2009; Liu et al., 2018). This situation mimics prevailing functional cholestasis without severe hepatocyte injury. TR rats displayed hyperbilirubinemia, which was further exacerbated by ethinylestradiol treatment (Figure 1F). Altogether, Mrp2 deficiency increases liver injury and causes severe hyperbilirubinemia in estrogen-induced cholestasis.

### Loss of Mrp2 Impairs Biliary Secretion of BAs

**Figure 2:**
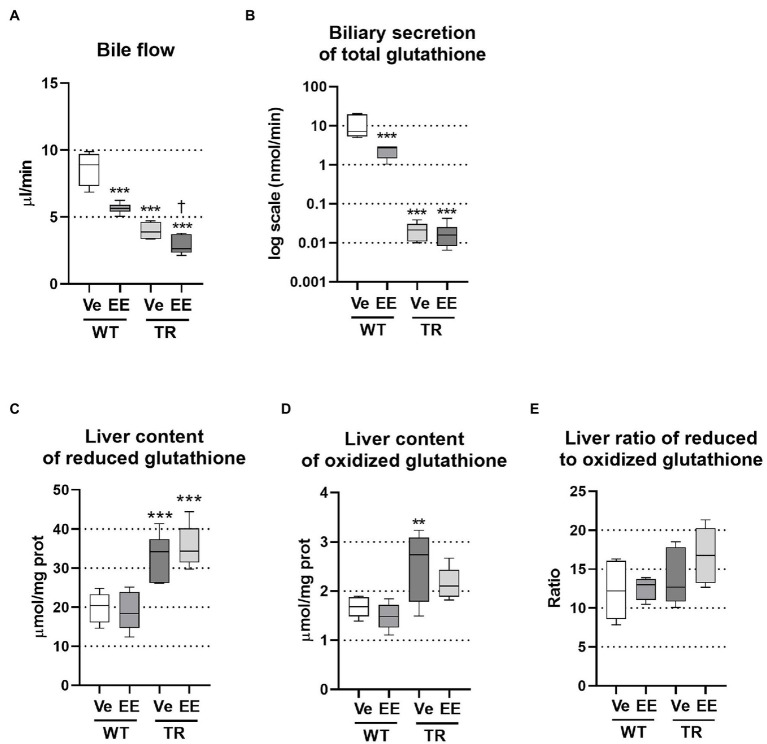
Ethinylestradiol administration reduced bile flow in WT and TR animals. Experimental groups: WT-Ve, WT rats receiving vehicle; WT-EE, WT rats receiving ethinylestradiol; TR-Ve, TR rats receiving vehicle; and TR-EE, TR rats receiving ethinylestradiol. **(A)** Bile flow rate. **(B)** Biliary secretion of glutathione. **(C,D)** Reduced and oxidized glutathione was measured in liver lysates and normalized to protein content. **(E)** Ratio of reduced to oxidized glutathione in the liver. Data are presented as medians, with boxes and whiskers representing the interquartile range and 5th-95th percentiles, respectively. Significance: ^**^p < 0.01, ^***^p < 0.001 compared to vehicle-treated WT rats; ^†^*p* < 0.05 compared to vehicle-treated TR rats.

Ethinylestradiol administration is a well-established rodent model of intrahepatic cholestasis associated with decreased bile secretion. The ability of ethinylestradiol to alter biliary secretion of bile acids was previously questioned in the Mrp2-deficient rats when ethinylestradiol was applied over 3 days (Koopen et al., 1998). Therefore, we applied ethinylestradiol over a 5-day period, which significantly reduced bile flow in both WT and TR animals (Figure 2A). Bile is formed by active biliary secretion of BAs (BA-dependent bile flow) and glutathione (major component of BA-independent bile flow). As expected, the biliary secretion of glutathione was significantly reduced in ethinylestradiol-treated WT rats and vehicle-treated TR group when compared to WT controls (Figure 2B). Ethinylestradiol administration did not have any additional effect on biliary glutathione secretion in TR rats when compared to vehicle treatment. Both TR groups also showed increased liver content of reduced glutathione (Figure 2C). Ethinylestradiol did not change liver reduced glutathione content in either group. Oxidized glutathione form was significantly increased only in vehicle treated TR rats (Figure 2D). Liver ratio of reduced to oxidized form of glutathione was not significantly modified either by ethinylestradiol or Mrp2 deficiency (Figure 2E).

**Figure 3:**
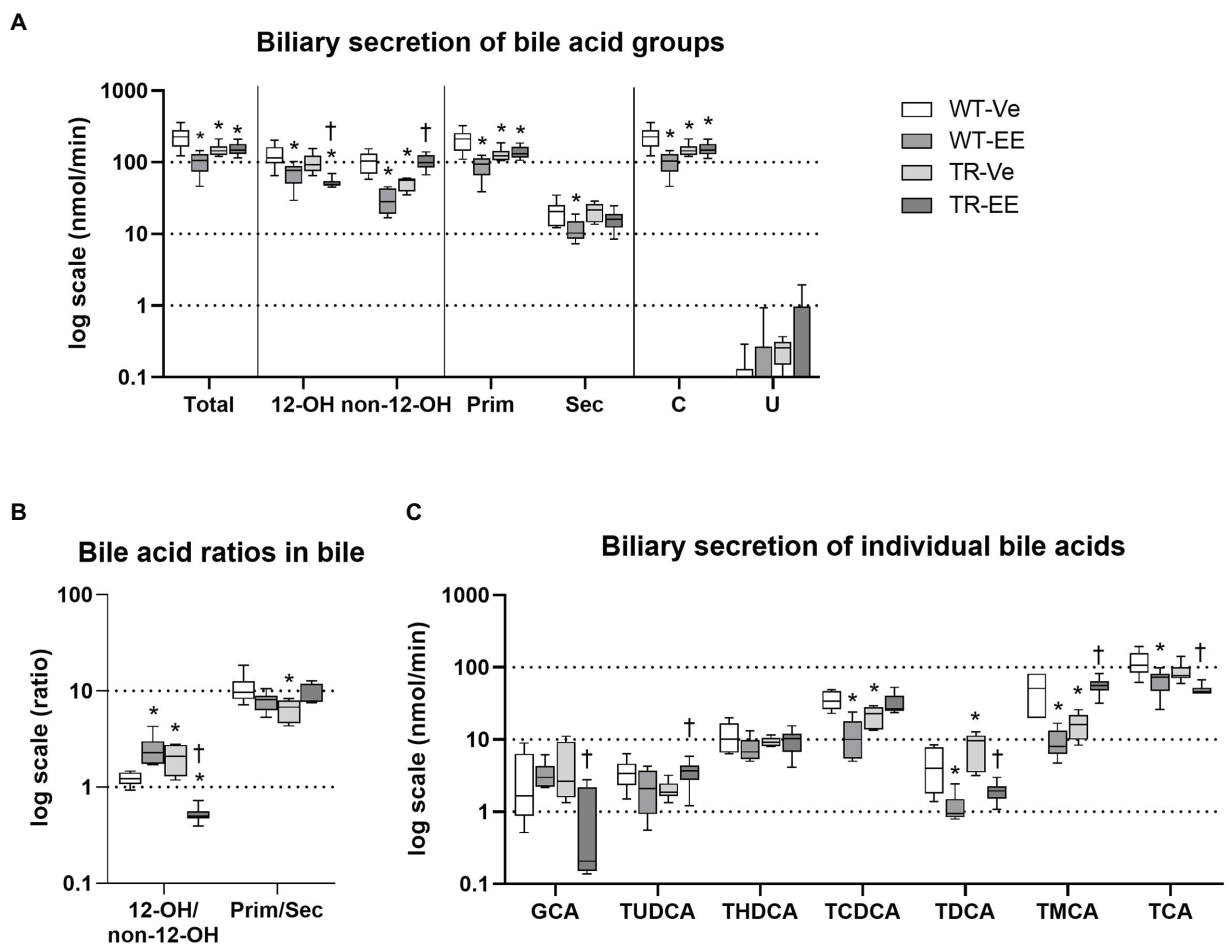
Ethinylestradiol administration reduced biliary secretion of bile acids (BAs) in WT and TR animals. Experimental groups: WT-Ve, WT rats receiving vehicle; WT-EE, WT rats receiving ethinylestradiol; TR-Ve, TR rats receiving vehicle; and TR-EE, TR rats receiving ethinylestradiol. **(A)** Biliary secretion of different types of BAs. **(B)** Biliary ratios of essential bile acid groups. **(C)** Biliary secretion of individual bile acids. Data are presented as medians, with boxes and whiskers representing the interquartile range and 5th-95th percentiles, respectively. Significance: ^*^p < 0.1 compared to vehicle-treated WT rats; ^†^*p* < 0.05 compared to vehicle-treated TR group. BA: 12α-hydroxylated (12-OH), non-12α-hydroxylated (non-12-OH), primary (Prim), secondary (Sec), conjugated (C), glycocholic acid (GCA), ursodeoxycholic acid (UDCA), hyodeoxycholic acid (HDCA), chenodeoxycholic acid (CDCA), deoxycholic acid (DCA), muricholic acid (MCA), and cholic acid (CA), with taurine conjugates TUDCA, taurohyodeoxycholic acid (THDCA), taurochenodeoxycholic acid (TCDCA), taurodeoxycholic acid (TDCA), tauro-muricholic acid (TMCA), and taurocholic acid (TCA).

Analysis of BAs in the bile showed a significant reduction in their net biliary secretion in ethinylestradiol treated WT rats, reflecting the reduction in 12α-hydroxylated, non-12α-hydroxylated, primary, secondary, and conjugated bile acids (Figure 3A). We have detected very low secretion of unconjugated BAs into bile in either group. Biliary secretion of total bile acids was also reduced in vehicle-treated TR rats due to reduced non-12α-hydroxylated, primary, conjugated bile acids. Administration of ethinylestradiol to TR rats did not further reduce biliary secretion of total bile acids but it reduced secretion of 12α-hydroxylated and increased that of non-12α-hydroxylated bile acids compared to vehicle-treated TR rats (Figure 3A). As a result, ratio of 12α-hydroxylated to non-12α-hydroxylated bile acids was increased in ethinylestradiol-administered WT rats, and in vehicle-administered TR rats, while it decreased in ethinylestradiol administered TR rats (Figure 3B). The biliary ratio of primary to secondary bile acids was reduced only in vehicle-treated TR rats compared to vehicle-treated WT group (Figure 3B). Significant discrepancies were noted in biliary secretion of individual bile acids (Figure 3C). Ethinylestradiol reduced biliary secretion of taurochenodeoxycholic acid (TCDCA), taurodeoxycholic acid (TDCA), tauro-muricholic acid (TMCA), and taurocholic acid (TCA) in WT rats. Mrp2 deficiency in vehicle-treated TR rats led to reduced biliary secretion of TCDCA and TMCA, and increased secretion of TDCA. Ethinylestradiol-treated TR rats had reduced biliary secretion of glycocholic acid (GCA), TDCA, and TCA, and increased secretion of TUDCA and TMCA compared to control TR rats. These data suggest that Mrp2 deficit indeed impairs biliary secretion of bile acids and their spectra.

### Intestinal Reabsorption of BAs Is Reduced in Mrp2-Deficient TR Rats

**Figure 4:**
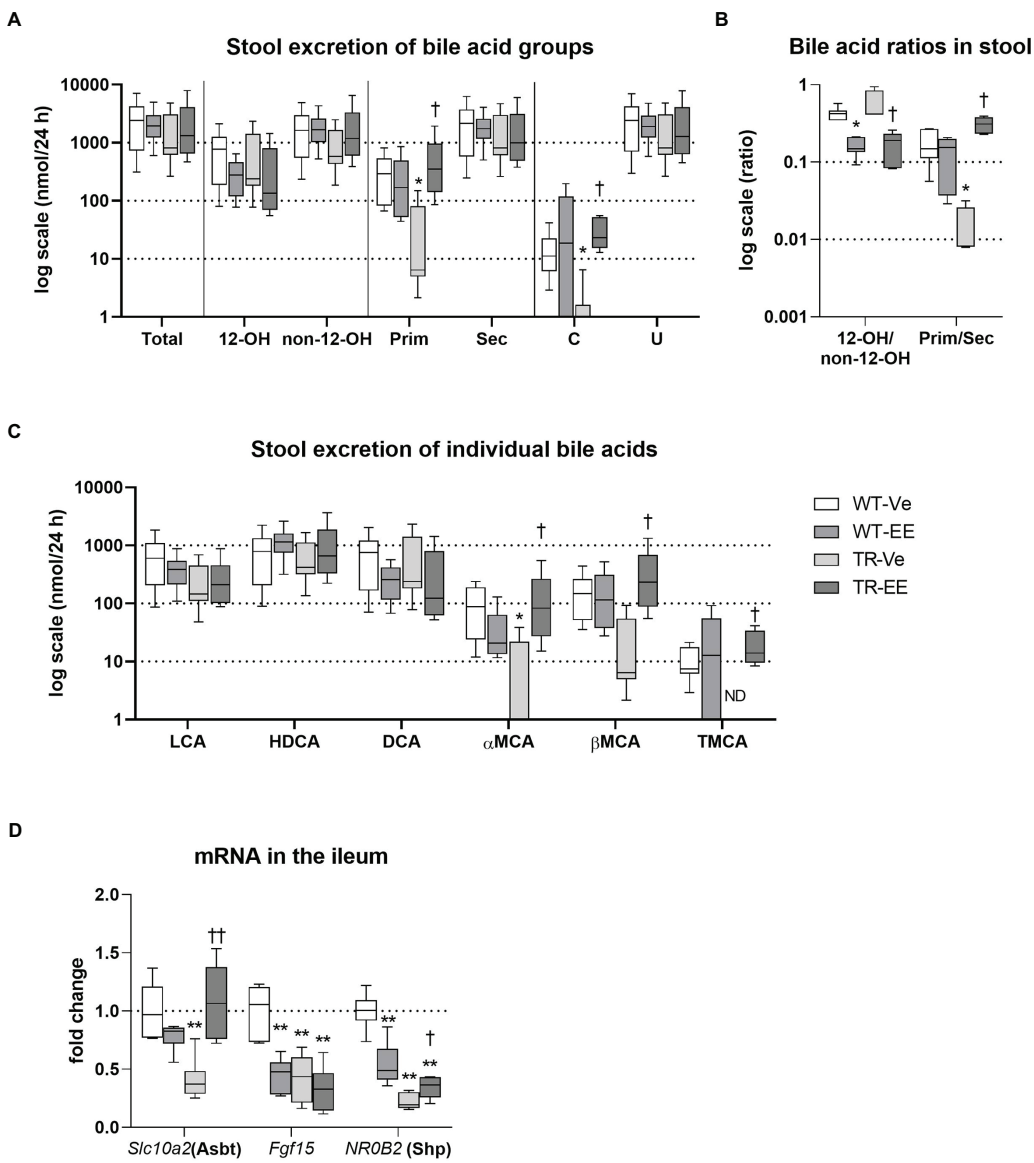
Intestinal homeostasis of bile acids. Experimental groups: WT-Ve, WT rats receiving vehicle; WT-EE, WT rats receiving ethinylestradiol; TR-Ve, TR rats receiving vehicle; and TR-EE, TR rats receiving ethinylestradiol. BAs were analyzed in the feces collected over 24 h. **(A)** Stool excretion of different types of bile acids. **(B)** Ratios of essential bile acid groups in stool. **(C)** Stool excretion of individual bile acids. **(D)** The mRNA expression levels of essential BA transporter and regulators were analyzed in the ileum. Data are presented as median values, with boxes and whiskers representing the interquartile range and 5th-95th percentiles, respectively. Significance: ^*^p < 0.05 and ^**^p < 0.01 compared to vehicle-treated WT rats. ^†^*p* < 0.05 and ^††^*p* < 0.01 compared to vehicle-treated TR group. BAs are presented as follows: 12-OH, non-12-OH, Prim, Sec, C, unconjugated (U), lithocholic acid (LCA), HDCA, DCA, α muricholic acid (αMCA), β muricholic acid (βMCA), and TMCA.

Bile acids delivered to the intestine *via* bile are metabolized by intestinal microbiota and mainly reabsorbed in the ileum. Reduced reabsorption of BAs from the intestine is one of the compensatory reactions of organism to cholestasis. Reduced reabsorption can be identified as retained stool excretion of BAs, while BA biliary secretion is reduced. This was indeed detected in WT rats administered with ethinylestradiol and in both TR groups (Figure 4A). Group analysis of BAs revealed reduced stool excretion of primary and conjugated BAs in vehicle-treated TR rats; and both these changes were restored by administration of ethinylestradiol (Figure 4A). Ethinylestradiol significantly reduced ratio of 12α-hydroxylated/non-12α-hydroxylated BAs in both WT and TR rats when compared with corresponding vehicle-treated groups (Figure 4B). Vehicle-treated TR rats had also significantly reduced the primary to secondary BA ratio compared with WT animals. This ratio was increased in TR animals by ethinylestradiol administration compared to vehicle-administered TR (Figure 4B). Stool excretions of individual BAs were modified only in TR groups (Figure 4C). Vehicle-treated TR rats showed significant decrease in stool excretion of TMCA and aMCA. Addition of ethinylestradiol significantly increased fecal content of aMCA, bMCA, and TMCA in TR rats (Figure 4C). The altered reabsorption of BAs, especially FXR agonists such as CDCA, into ileum enterocytes changed the expression of molecules involved in BA homeostasis (Figure 4D). The expression of *Slc10a2* (Asbt protein), the apical transporter for BA reabsorption, was significantly reduced in vehicle-treated TR group when compared with vehicle-treated WT rats. The *Slc10a2* also showed tendency toward reduction (*p* = 0.075) in ethinylestradiol-administered WT rats compared with vehicle-treated WT. The major FXR target genes, *Fgf15* and *NR0B2* (Shp) were both suppressed in ethinylestradiol-treated WT rats and in both TR groups compared with vehicle-administered WT rats. Ethinylestradiol significantly increased *Slc10a2* a *NR0B2* expression in Mrp2-deficient rats relative to vehicle administered TR group. These results indicate reduced intestinal reabsorption of BAs in WT and TR rats with reduced FXR receptor activation in ileum of these groups.

### Ethinylestradiol Further Increases Already Raised Plasma BA Concentrations in TR Rats

**Figure 5:**
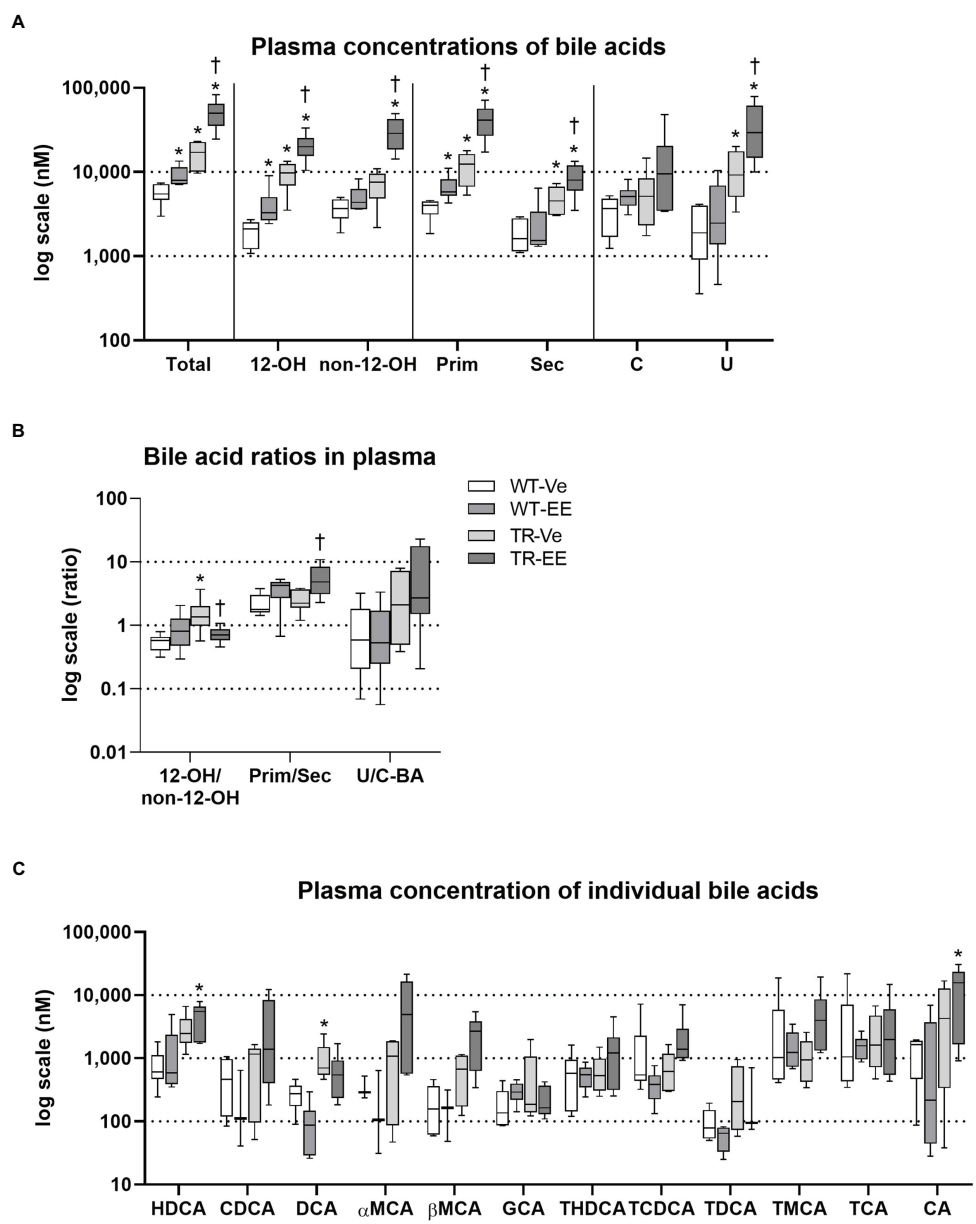
Ethinylestradiol administration increased plasma concentrations of bile acids in WT and TR animals. Experimental groups: WT-Ve, WT rats receiving vehicle; WT-EE, WT rats receiving ethinylestradiol; TR-Ve, TR rats receiving vehicle; and TR-EE, TR rats receiving ethinylestradiol. **(A)** Types of bile acids measured in plasma. **(B)** Ratios of essential bile acid groups in plasma. **(C)** Concentrations of individual bile acids in the plasma. Data are presented as median values, with boxes and whiskers representing the interquartile range and 5th-95th percentiles, respectively. Significance: ^*^p < 0.05 compared to vehicle-treated WT rats; ^†^*p* < 0.05 compared to vehicle-treated TR group. BAs are presented as follows: 12-OH, non-12-OH, Prim, Sec, C, U, HDCA, CDCA, DCA, αMCA, βMCA, GCA, THDCA, TCDCA, TDCA, TMCA, TCA, and CA.

Liquid chromatography–mass spectrometry (LC–MS) analysis of BA spectra in plasma identified significantly increased total BA concentrations in ethinylestradiol-treated WT rats, when compared to vehicle-treated WT group (Figure 5A). This increase was due to an increase in 12α-hydroxylated, primary bile acids. Mrp2 deficit in TR rats also led to a significant increase in total plasma BA concentrations due to an increase in 12α-hydroxylated, primary, secondary but also unconjugated BAs relative to control WT rats. Administration of estrogen to TR rats further increased net plasma BAs by raising 12α-hydroxylated, non-12α-hydroxylated, primary, secondary, and unconjugated BA concentrations. Vehicle-treated TR rats showed increased 12α-hydroxylated/non-12α-hydroxylated ratio compared to vehicle-treated WT group (Figure 5B). In contrast, ethinylestradiol reduced 12α-hydroxylated/non-12α-hydroxylated, and increased primary/secondary BA ratios in TR rats (Figure 5B). Individual bile acids showed just a tendency for increased plasma concentrations in cholestatic groups (Figure 5C). Only the plasma concentration of deoxycholic acid (DCA) was markedly increased in vehicle-treated TR rats compared to control WT, raising thus the 12α-hydroxylated/non-12α-hydroxylated BA ratio. These results indicate accumulation of BAs in plasma of Mrp2-deficient TR rats, which is further worsened by ethinylestradiol.

### Executive Pathways of BA Enterohepatic Recycling Are Impaired in TR Rats

**Figure 6:**
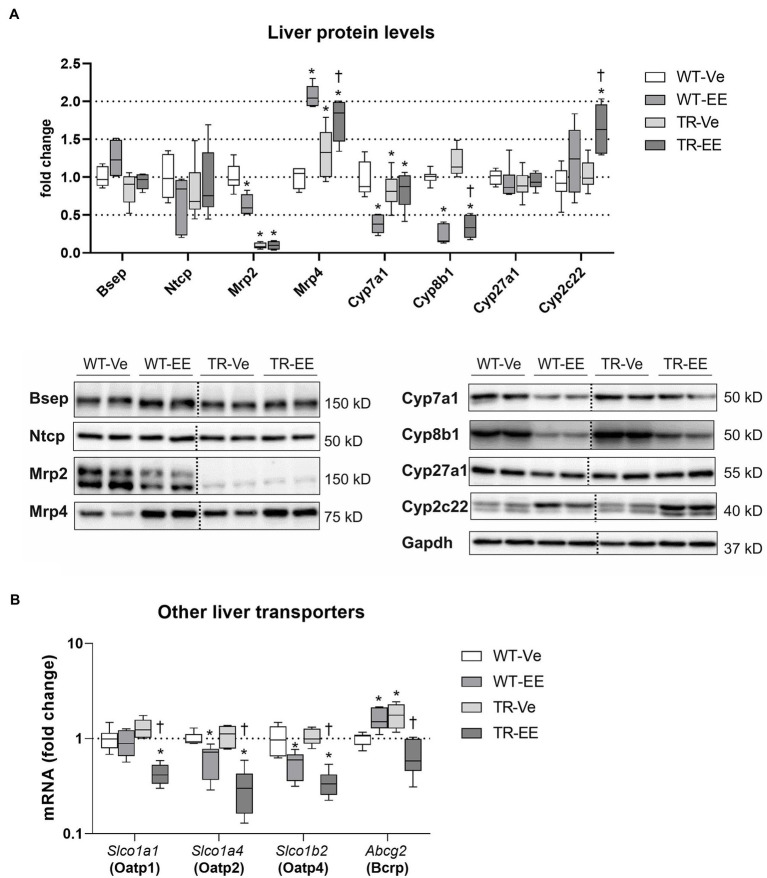
Effect of ethinylestradiol and Mrp2 deficiency on the mRNA and protein expression of bile acid-related genes in the liver. Experimental groups: WT-Ve, WT rats receiving vehicle; WT-EE, WT rats receiving ethinylestradiol; TR-Ve, TR rats receiving vehicle; and TR-EE, TR rats receiving ethinylestradiol. **(A)** The protein expression of crucial bile acid synthetizing and transporting molecules in the liver. Dotted line shows where irrelevant bands were omitted. **(B)** The gene expression of alternative hepatocyte transporters for bile acids. Protein and mRNA levels were normalized to Gapdh expression. Data are presented as medians, with boxes and whiskers representing the interquartile range and 5th-95th percentiles, respectively. Protein and mRNA levels were normalized to Gapdh expression. Significance: ^*^p < 0.05, compared to vehicle-treated WT rats; ^†^*p* < 0.05 compared to vehicle-treated TR group.

To unravel mechanisms of increased plasma BA concentrations in Mrp2-deficient TR rats, we analyzed major enzymes and transporters necessary for BA enterohepatic recycling (Figure 6A). As expected, ethinylestradiol administration to WT animals predictably reduced liver protein expression of Na^+^-taurocholate cotransporting polypeptide (Ntcp; major basolateral transporter for uptake of BAs to hepatocytes), Mrp2, cholesterol 7α-hydroxylase (Cyp7a1; the rate-limiting enzyme for BA synthesis), and sterol 12α-hydroxylase (Cyp8b1; the crucial enzyme for 12α-hydroxylated BA synthesis), and upregulated multidrug resistance-associated protein 4 (Mrp4; transporter responsible for the efflux of BAs from hepatocytes to plasma). As expected, Mrp2-deficient TR rats had negligible protein expression of Mrp2. In addition, these animals had downregulation of Cyp7a1, and upregulation Mrp4 proteins. Ethinylestradiol administration to TR rats downregulated Cyp8b1 and markedly induced Mrp4 efflux protein (Figure 6A). Other transporters for BAs such as *Slco1a1*, *Slco1a4*, *Slco1b2*, and *Abcg2* were reduced by ethinylestradiol especially in TR group compared to vehicle-administered controls (Figure 6B). Interestingly, the expression of *Slco1a1* and *Abcg2* was unchanged or even increased in WT-EE rats compared to the WT-Ve group indicating different patterns of regulation.

### Nrf2 Was the Major Regulatory Pathway of BA Metabolomics Activated in TR Rats

**Figure 7:**
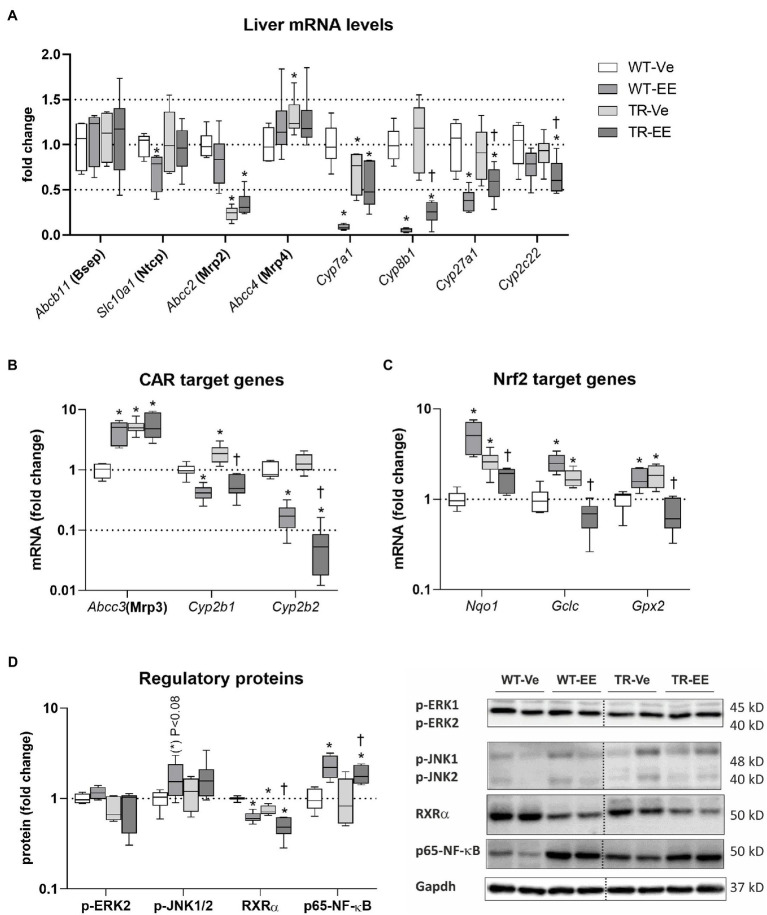
Effect of ethinylestradiol and Mrp2 deficiency on the mRNA or protein expression of pathways regulating bile acid homeostasis in the liver. Experimental groups: WT-Ve, WT rats receiving vehicle; WT-EE, WT rats receiving ethinylestradiol; TR-Ve, TR rats receiving vehicle; and TR-EE, TR rats receiving ethinylestradiol. **(A)** The mRNA expression of crucial bile acid synthetizing and transporting molecules in the liver. **(B)** The expression of genes regulated by constitutive androstane receptor (CAR). **(C)** The expression of Nuclear factor erythroid 2-related factor 2 (Nrf2) target genes. **(D)** Protein expression of key intracellular signaling molecules involved in the regulation of bile acid metabolomics. Protein and mRNA levels were normalized to Gapdh expression. Dotted line shows where irrelevant bands were omitted. Data are presented as medians, with boxes and whiskers representing the interquartile range and 5th-95th percentiles, respectively. Significance: ^*^p < 0.05, compared to vehicle-treated WT rats; ^†^*p* < 0.05 compared to vehicle-treated TR group.

Multiple pathways are involved in the regulation of BA homeostasis (Chiang and Ferrell, 2020). Therefore, we screened the principal ones associated with estrogen-induced cholestasis. Compared to vehicle-treated controls, ethinylestradiol activated FXR-Shp signaling in the liver of WT rats, as apparent from the repressed mRNA of *Cyp7a1*, *Cyp8b1*, *Cyp27a1*, and *Slc10a1* (Ntcp; Figure 7A). *Abcb11* (Bsep), *Abcc2* (Mrp2), and *Abcc4* (Mrp4) were not regulated by ethinylestradiol at the transcriptional level (Figure 7A). Mrp2 deficiency in vehicle-treated TR rats was associated with reduced mRNA expression of *Cyp7a1* and induced *Abcc3* and *Abcc4* (Figures 7A,B). Administration of ethinylestradiol to TR rats reduced mRNA expression of *Cyp8b1*, *Cyp27a1*, and *Cyp2c22* without effect on mRNA of other transporters and enzymes.

The mRNA expression of constitutive androstane receptor (CAR) target genes such as *Cyp2b1/2* was reduced in ethinylestradiol administered groups compared with respective vehicle administered animals (Figure 7B). Another CAR target, *Abcc3*, was induced by ethinylestradiol in WT group, but unchanged in TR rats. CAR targets were induced in vehicle-treated TR group. Ethinylestradiol-induced Nuclear factor erythroid 2-related factor 2 (Nrf2) target gene expression such as *Nqo1*, *Gclc*, and *Gpx2* in WT animals (Figure 7C). Similar induction was detected in vehicle-treated TR rats relative to WT group. In contrast, administration of ethinylestradiol to TR animals reduced mRNA expression of Nrf2 target genes compared to vehicle-treated TR rats. Intracellular MAP kinases, RXRa, and Nuclear factor NF-kappa-B (NF-kB) are another intracellular regulators of bile acid metabolism involved in the response of liver to ethinylestradiol administration. Indeed, tendency toward induction in p-JNK1/2 (*p* < 0.08), significantly induced p65-NF-kB and reduced RXRa were detected in WT rats administered with ethinylestradiol (Figure 7D). The RXRa was reduced in vehicle-treated TR rats compared with control WT group. Ethinylestradiol administration to TR rats caused further downregulation of RXRa and upregulation of p65-NF-kB relative to vehicle-treated TR animals. These results indicate that there are significant differences in ethinylestradiol-mediated regulation of BA homeostasis between WT and Mrp2-deficient rats.

## Discussion

The major finding of our study is that Mrp2-deficient animals show increased plasma concentrations of BAs and are sensitive to ethinylestradiol-induced cholestatic insult. Measurement of individual BAs in Mrp2-deficient animals was previously performed in one study, where the authors presented only relative peak areas standardized to D4-taurocholic acid using LC–MS analysis. This analysis showed a 1.6–62.1-fold increase in peak areas of plasma BAs in Mrp2-deficient animals compared to WT animals (Aoki et al., 2011). However, the accuracy of such a standardization method may be altered by different chemical characteristics of individual BAs. Indeed, our LC–MS analysis of absolute BA concentrations in plasma with separate calibrators showed an increased concentration of total BAs in plasma. This was the result of an overall tendency toward an increase for the majority of individual BAs, with the increase in DCA, a secondary 12α-hydroxylated bile acid (12-OH BA), reaching statistical significance. These data suggest a change at the level of net BA transport and synthesis of secondary BAs in Mrp2-deficient TR rats. Importantly, the shift of the plasma BA spectrum toward more lipophilic DCA may imply an increased risk of systemic toxicity.

Our vehicle-treated TR rats showed an expected reduction in BA-independent bile flow *via* reduction in Mrp2-mediated biliary secretion of glutathione. Mrp2 also transports glucuronide- and sulfate-conjugates of BAs, especially TCDCA and TLCA (Kuipers et al., 1988; Takikawa et al., 1991; Akita et al., 2001). Previously, in Mrp2-deficient animals, net biliary secretion of BAs was not changed (Verkade et al., 1993) and concentrations of unconjugated and glycine/taurine-conjugated BAs were not different after administration of radiolabeled ^14^C-CDCA (Takikawa et al., 1991). However, at the time of these analyses, a sensitive method for BA detection was not available. Indeed, our results achieved by highly sensitive LC–MS analysis showed that biliary secretion of total BAs was reduced at the expense of monoconjugated TCDCA, TMCA, while secretion of TDCA was increased. We identified several factors which may contribute to this effect in Mrp2-deficient TR rats. First, BA synthesis in hepatocytes was reduced *via* repressed Cyp7a1, and BAs underwent increased efflux from hepatocytes to plasma *via* induced multidrug resistance-associated protein 3 (Mrp3) and Mrp4, thus reducing disposition of BAs for biliary secretion. This is consistent with previously described induction of Mrp3 and Mrp4 in Mrp2-deficient rats (Oswald et al., 2006; Gavrilova et al., 2007). As stated previously, Mrp3 level correlates with BA return to plasma (Akita et al., 2002), and Mrp4 transporter may contribute to this effect. Second, stool excretion of BAs was not changed despite the reduced BA intestinal delivery due to reduced BA biliary secretion. This indicates that BA reabsorption is reduced in the ileum, consistent with reduced expression of Asbt, a major absorption transporter for BAs. Therefore, reduced ileum expression of FXR target genes, such as Shp and Fgf15, may be a consequence of decreased reabsorption of FXR agonistic BAs, which was indeed detected for TCDCA. Interestingly, increased biliary secretion was noticed for TDCA. Coupled with unchanged stool excretion of DCA, this indicated that reabsorption of this BA in the ileum is increased. We may only speculate that DCA was reabsorbed by passive diffusion in the large intestine (Chiang and Ferrell, 2020). This may explain increased concentrations of DCA in the plasma of TR rats. Such a shift in the spectrum of BAs reduced the primary to secondary BA ratio in bile and stool of TR rats. It suggests increased conversion of primary BAs into secondary BAs by modified intestinal microbiota as a result of the altered flux of Mrp2 substrates, including BAs, from the liver to the intestine *via* bile. These findings indicate that Mrp2-deficient organism may be predisposed to a more significant increase in plasma BA concentrations upon hormonal cholestatic insult.

Analysis of BA regulatory pathways revealed that liver changes in our Mrp2-deficient rats may be related to the activation of Nrf2 as reflected by increased expression of its target genes, such as *Nqo1*, *Gclc*, and *Gpx2*. Nrf2 plays a critical role in regulating the transcription of cytoprotective genes during oxidative stress. Recently, it was shown that Nrf2 activation induces Mrp3 and Mrp4, represses Cyp7a1, decreases BA concentrations in the liver, and increases BAs in plasma (Aleksunes et al., 2008; Xu et al., 2010; Aleksunes and Klaassen, 2012; Liu et al., 2021), which was also apparent in our study. In addition, repression of Asbt in ileum enterocytes detected in our study was also seen upon Nrf2 activation (Zhang et al., 2020). Activation of Nrf2 may contribute to glutathione accumulation in TR rats due to activation of glutathione synthetizing enzymes in addition to the reduced glutathione biliary secretion due to Mrp2 deficiency. Interestingly, accumulating reduced glutathione and bilirubin provide significant antioxidative activity in the liver of Mrp2-deficient rats, yet Nrf2 is activated. This may be a consequence of CAR activation (Rooney et al., 2019) by accumulated bilirubin, which is a known CAR agonist (Vítek, 2020). Indeed, we observed marked induction of CAR target genes Abcc3, and Cyp2b1. Therefore, one potential mechanism of observed changes in BA homeostasis in TR rats may be bilirubin-mediated activation of CAR-Nfr2 pathway. TR rats also showed decreased RXRa, a versatile dimerization partner (Lefebvre et al., 2010) required for the formation of active heterodimers with orphan nuclear receptors involved in the regulation of BA metabolomics such as FXR (Garcia et al., 2018). Other regulatory pathways seem to have a minor role in TR rats. Fgf15 released from the ileum is a major regulator BA synthesis by suppressing Cyp7a1 expression in the liver through activation of the Fgfr4-JNK (c-Jun-N-terminal kinase) pathway (Chiang and Ferrell, 2020). Reduction of Fgf15 without activation of JNK excludes the contribution of Fgf15 to the observed Cyp7a1 reduction.

The major mechanism responsible for estrogen-induced cholestasis is the activation of ERa receptor with consequent transcriptional repression of basolateral uptake transporters for BAs such as *Slc10a1* (Ntcp), and BA synthetic enzymes such as Cyp7a1 and Cyp8a1 (Geier et al., 2003, 2007; Yamamoto et al., 2006). Ethinylestradiol reduces transactivation of these molecules by decreasing regulatory hepatocyte nuclear factor 1 (HNF1), and RXR: RAR factors, either directly or by induction of inflammatory reactions that activate several intracellular stress pathways including NF-kB, and JNK (Geier et al., 2007). In agreement, our estrogen-treated WT rats showed a decrease in Ntcp, Oatp, Cyp7a1, Cyp8b1, and RXRa and activation of JNK and NF-kB. Similarly to previous results with ethinylestradiol-induced cholestasis, we also detected posttranscriptional reduction of Mrp2 (Trauner et al., 1997; Lee et al., 2000; Wagner et al., 2003; Ruiz et al., 2007; Cermanova et al., 2015), Mrp4 upregulation (Faradonbeh et al., 2021), and unchanged Bsep protein expression (Ruiz et al., 2007). Cholestasis after ethinylestradiol is typically associated with ERa-JNK-c-JUN mediated (Ruiz et al., 2013) upregulation of Mrp3 basolateral efflux transporter for BAs (Hirsova et al., 2013; Muchova et al., 2015), which all were also reproduced in our study. These mechanisms impede the transcellular transport of BAs and increase their concentrations in plasma. Furthermore, reduced biliary secretion of BAs in ethinylestradiol-treated rats was coupled with unchanged fecal BA elimination. This indicates that reduced BA reabsorption in the intestine represents a compensatory mechanism to restore BA homeostasis during cholestasis (Zhang et al., 2018). In agreement, reduced uptake of FXR agonistic BAs, such as CDCA and DCA, in the ileum led to reduced expression of target genes such as *NR0B2* (Shp) and *Fgf15* as also previously reported (Hirsova et al., 2013; Faradonbeh et al., 2021).

Few earlier studies analyzed estrogen-induced cholestasis in Mrp2-deficient rats. Koopen et al. (1998) showed reduced bile flow in Mrp2-deficient rats after 3-day ethinylestradiol treatment, although biliary secretion of glutathione, bilirubin, and net BAs was not changed. Two other studies with Mrp2-deficient rats failed to find the immediate effect of a single estrogen dose on the bile flow (Huang et al., 2000) or Bsep-mediated taurocholate uptake to canalicular membrane vesicles (Stieger et al., 2000). This was ascribed to the absence of Mrp2-mediated biliary secretion of cholestatic metabolites of ethinylestradiol, such as ethinylestradiol-17-α/β-glucuronide, that consequently block Bsep from the luminal site (Huang et al., 2000; Stieger et al., 2000). However, our 5-day regimen of ethinylestradiol reliably induced cholestasis in the Mrp2-deficient rats. We detected an additional increase of plasma BA concentrations, confirming an increased sensitivity of Mrp2-defective organism to estrogen. Such sensitivity may result from increased exposure of organisms to ethinylestradiol due to altered ethinylestradiol-glucuronide excretion *via* Mrp2 (Zamek-Gliszczynski et al., 2011). Increased plasma BAs were consistent with marked increase in Mrp4-mediated export of BAs back to plasma. In addition, *Slco1a1* and *Abcg2* have been suggested as important hepatocyte transporters for the uptake and secretion of BAs, respectively (Blazquez et al., 2012; Tanaka et al., 2012; Zhang et al., 2012; Jiang et al., 2020). Significant reduction of *Slco1a1* and *Abcg2* in ethinylestradiol administered Mrp2-deficient rats compared with vehicle-treated deficient group contrasts with absence (*Slco1a1*) or even induction (*Abcg2*) of these transporters in ethinylestradiol-treated WT group. However, unchanged biliary secretion of bile acids between Mrp2-deficient groups suggests that the contribution of *Abcg2* to observed changes in BA metabolomics may not be significant. Taking together, reduction of *Slco1a1* expression by ethinylestradiol in Mrp2-deficient rats may contribute to increased BA plasma concentrations in this group *via* decreased BAs uptake into hepatocytes. A compensatory reaction to increased plasma BAs likely involved a decreased expression of Cyp8b1, the rate-limiting enzyme for the synthesis of 12-OH BAs, followed by a generally reduced 12-OH/non-12-OH BA ratio in plasma, bile, and stool. Unlike Cyp7a1, the Cyp8b1 is mainly regulated by liver FXR (Chiang and Ferrell, 2020). Reduced expression of Cyp8b1 may therefore reflect the inhibitory effect of ethinylestradiol on FXR and its cofactor RXRa. In contrast, ethinylestradiol posttranscriptionally induced expression of Cyp2c22, a rat orthologue synthetizing primary muricholic acid (MCA), leading to increased TMCA in bile and stool, and contributing to increased primary/secondary BA ratio in the stool. Unlike in WT rats, ethinylestradiol consistently reduced Nrf2 activation in TR animals by unknown mechanisms.

## Conclusion

Our findings showed marked modification of BA metabolomics and a significant increase in BA plasma concentrations in Mrp2-deficient rats. The mechanism responsible for this increase was likely related to the increased Mrp3 and Mrp4-mediated efflux of BAs from hepatocytes to plasma. Reduced BA biliary secretion and reabsorption revealed reduced enterohepatic recycling of BAs in Mrp2-deficient animals, except for increased disposition of secondary DCA. Possible alteration of intestinal microbiota in the Mrp2-deficient organism is therefore suggestive but requires further analysis. Accumulation of more lipophilic secondary BAs may threaten the organism with their increased toxicity. Ethinylestradiol administration to Mrp2-deficient TR rats further increased concentrations of BAs in plasma by upregulation of liver Mrp4 efflux pump, and reduced *Slco1a1* uptake transporter. Taken together, our results present mechanisms that may explain the increased sensitivity of Mrp2-deficient organisms to estrogen-induced cholestasis. Bile acid monitoring is therefore highly desirable in pregnant women with conjugated hyperbilirubinemia for early detection of ICP.

## Data Availability Statement

The original contributions presented in the study are included in the article/supplementary material, further inquiries can be directed to the corresponding author.

## Ethics Statement

The project was reviewed and approved by the Animal Welfare Bodies of the Faculty of Medicine in Hradec Kralove and the Ministry of Education, Youth and Sports of the Czech Republic.

## Author Contributions

FAF: methodology, investigation, and writing—original draft. HL, JC, MH, and MU: investigation and methodology. ZN: software. PH: writing—review and editing. PP: validation. SM: supervision, project administration, resources, funding acquisition, and writing—review and editing. All authors contributed to the article and approved the submitted version.

## Funding

The project was supported by grants Progres Q40/05, SVV 260543/2020, GAUK 5562/18, and GACR 19-14497S, and by the ERDF-Project PERSONMED No. CZ.02.1.01/0.0/0.0/16_048/0007441 and ESF-Project “International mobility of RTAS ChU” No. CZ.02.1.01/0.0/0.0/17_048/0007421. PH received support from the National Institute of Diabetes and Digestive and Kidney Diseases (NIDDK) of the National Institutes of Health (NIH) under the Award Number R01DK130884.

## Conflict of Interest

The authors declare that the research was conducted in the absence of any commercial or financial relationships that could be construed as a potential conflict of interest.

## Publisher’s Note

All claims expressed in this article are solely those of the authors and do not necessarily represent those of their affiliated organizations, or those of the publisher, the editors and the reviewers. Any product that may be evaluated in this article, or claim that may be made by its manufacturer, is not guaranteed or endorsed by the publisher.

## Figures and Tables

**Table:** 

12-OH	12α-hydroxylated bile acid
Abcc2	ATP-binding cassette transporter sub-family C-member 2
Abcg2	ATP-binding cassette transporter sub-family G-member 2
ALT	Alanine aminotransferase
ANOVA	Analysis of variance
Asbt	Ileal sodium/bile acid cotransporter
AST	Aspartate aminotransferase
ATP	Adenosine triphosphate
BA	Bile acid
BADF	Bile acid-dependent bile flow
BAIF	Bile acid-independent bile flow
BE	Biliary excretion
Bsep	Bile salt export pump
CAR	Constitutive androstane receptor
CDCA	Chenodeoxycholic acid
Cyp27a1	Sterol 27-hydroxylase
Cyp2b1	Cytochrome P450 family 2, subfamily b, polypeptide 1
Cyp2c22	Cytochrome P450, family 2, subfamily c, polypeptide 22
Cyp7a1	Cholesterol 7α-hydroxylase
Cyp8b1	Sterol 12α-hydroxylase
CAD5	D5-cholic acid
DCA	Deoxycholic acid
EE	Ethinylestradiol
ERK	Extracellular signal-regulated kinase
Fgf15	Fibroblast growth factor 15
FXR	Farnesoid X receptor
Gapdh	Glyceraldehyde 3-phosphate dehydrogenase
GCA	Glycocholic acid
Gclc	Glutamate-cysteine ligase catalytic subunit
Gpx2	Glutathione peroxidase 2
GSH	Reduced glutathione
GSSG	Oxidized glutathione
HPLC	High-performance liquid chromatography
ICP	Intrahepatic cholestasis of pregnancy
LCA	Lithocholic acid
LC–MS	Liquid chromatography–mass spectrometry
MCA	Muricholic acid
Mrp2	Multidrug resistance-associated protein 2
Mrp3	Multidrug resistance-associated protein 3
Mrp4	Multidrug resistance-associated protein 4
Non-12-OH	Non-12α-hydroxylated
Nqo1	NAD(P)H quinone dehydrogenase 1
Nrf2	Nuclear factor erythroid 2-related factor 2
Ntcp	Na^+^-taurocholate cotransporting polypeptide
P65 NF-kB	Nuclear factor NF-kappa-B p65 subunit
p-JNK	Phosphorylated c-Jun N-terminal kinases
PXR	Pregnane X receptor
qRT-PCR	Quantitative reverse transcription-PCR
RXRα	Retinoid X receptor alpha
SHP	Small heterodimer partner
TCA	Taurocholic acid
TCDCA	Taurochenodeoxycholic acid
TDCA	Taurodeoxycholic acid
TLCA	Taurolithocholic acid
TMCA	Tauromuricholic acid
TR rats	Mrp2-deficient rats
UDCA	Ursodeoxycholic acid
WT	Wild-type
